# CNVs and Chromosomal Aneuploidy in Patients With Early-Onset Schizophrenia and Bipolar Disorder: Genotype-Phenotype Associations

**DOI:** 10.3389/fpsyt.2020.606372

**Published:** 2021-01-12

**Authors:** Hojka Gregoric Kumperscak, Danijela Krgovic, Maja Drobnic Radobuljac, Nina Senica, Andreja Zagorac, Nadja Kokalj Vokac

**Affiliations:** ^1^Department of Pediatrics, University Medical Center Maribor, Maribor, Slovenia; ^2^Medical Faculty, University of Maribor, Maribor, Slovenia; ^3^Laboratory of Medical Genetics, University Medical Center Maribor, Maribor, Slovenia; ^4^Unit for Intensive Child and Adolescent Psychiatry, Center for Mental Health, University Psychiatric Clinic Ljubljana, Ljubljana, Slovenia; ^5^Medical Faculty, University of Ljubljana, Ljubljana, Slovenia

**Keywords:** early onset, schizophrenia, bipolar disorder, copy number variations, chromosomal aneuploidy

## Abstract

**Introduction:** Early-onset schizophrenia (EOS) and bipolar disorder (EOB) start before the age of 18 years and have a more severe clinical course, a worse prognosis, and a greater genetic loading compared to the late-onset forms. Copy number variations (CNVs) are an important genetic factor in the etiology of psychiatric disorders. Therefore, this study aimed to analyze CNVs in patients with EOS and EOB and to establish genotype-phenotype relationships for contiguous gene syndromes or genes affected by identified CNVs.

**Methods:** Molecular karyotyping was performed in 45 patients, 38 with EOS and seven with EOB hospitalized between 2010 and 2017. The exclusion criteria were medical or neurological disorders or IQ under 70. Detected CNVs were analyzed according to the standards and guidelines of the American College of Medical Genetics.

**Result:** Molecular karyotyping showed CNVs in four patients with EOS (encompassing the *PAK2, ADAMTS3*, and *ADAMTSL1* genes, and the 16p11.2 microduplication syndrome) and in two patients with EOB (encompassing the *ARHGAP11B* and *PRODH* genes). In one patient with EOB, a chromosomal aneuploidy 47, XYY was found.

**Discussion:** Our study is the first study of CNVs in EOS and EOB patients in Slovenia. Our findings support the association of the *PAK2, ARHGAP11B*, and *PRODH* genes with schizophrenia and/or bipolar disorder. To our knowledge, this is also the first report of a multiplication of the *ADAMTSL1* gene and the smallest deletion of the *PAK2* gene in a patient with EOS, and one of the few reports of the 47, XYY karyotype in a patient with EOB.

## Introduction

Schizophrenia and bipolar disorder are severe and chronic mental disorders with a profound effect on a person's life. While both disorders are very rare in childhood, they become more frequent during adolescence since the prevalence of both disorders in adulthood is around 1% (1, 2). Some clinical symptoms can be present in both schizophrenia and bipolar disorder, including impaired cognitive function, mood dysregulation, and psychotic symptoms (3). If schizophrenia or bipolar disorder becomes clinically manifested before the age of 18 years, the term early-onset schizophrenia (EOS) or early-onset bipolar disorder (EOB) is used (1, 2). Even though EOS and EOB lie on a continuum with schizophrenia and bipolar disorder in adults and the same diagnostic criteria are valid for all age groups, there are special difficulties in applying the adult criteria to children or adolescents. Symptoms at an early stage are less specific and also show remarkable overlap with several developmental disorders. The prognosis of EOS is worse than schizophrenia that starts in adulthood and EOS may have a higher genetic loading than adult-onset schizophrenia (2). Similarly, genetic loading may play a greater role in early-onset bipolar disorder compared to the late-onset form (4). The study of Toma et al. found that the genome-wide burden of likely gene-disruptive variants correlated significantly with the age of onset (*P* = 0.017), suggesting that a high disruptive variant burden may expedite symptom onset in a bipolar patient. This study showed that severe mutations (truncating alleles) are negatively correlated with early age of onset in bipolar disorder (5). Clinical studies have shown that EOB is more severe and homogeneous than other forms of bipolar disorder and is associated with a higher recurrence rate of mood episodes, higher rates of psychotic symptoms and comorbid conditions, and more frequent suicide attempts and neurocognitive impairments (6, 7).

Schizophrenia and bipolar disorder are highly polygenetic disorders with many different loci contributing to the development. Results from the latest studies suggest that the clinical overlap between schizophrenia and bipolar disorder results in part from their shared genetic liability (8, 9). There is also a genetic overlap with other neuropsychiatric disorders, such as autism spectrum disorder (ASD), intellectual disability (ID), and major depressive disorder (10). Rare copy number variations (CNVs) play an important role in the liability to schizophrenia (11). One of the most studied CNVs associated with schizophrenia is the 22q11.2 deletion, which has also been connected with other psychiatric disorders (12). The role of rare point variants in the risk of developing schizophrenia is still unclear (13, 14). It has been confirmed in many studies that the same common polygenetic variation contributes to the risk of both schizophrenia and bipolar disorder (3, 15). Studies that explored the associations of CNVs with schizophrenia and bipolar disorder have shown a higher burden of rare and large CNVs in patients with schizophrenia than in controls, including 1q21.1, 2p16.3, 3q29, 15q13.3, 16p11.2, 17q12, and 22q11.21 (16, 17) and that the effect of rare large CNVs on bipolar disorder seems less prominent (18–20). Several rare, large CNVs previously associated with schizophrenia have also been reported to contribute to the risk of bipolar disorder, including 3q29, 15q13.3, and 16p11.2 (21, 22). Chen et al. also found CNVs that might be bipolar disorder-specific, including 17q21.1, 9p21.3, and 9q21.13 (3).

The aim of the present study was to analyze genomic structural variations (CNVs) and chromosomal aneuploidies in young Slovenian patients suffering from severe mental disorders with an onset before the age of 18 years. Elucidation of the role of specific schizophrenia-associated genes affected by identified CNVs or contiguous gene syndromes is given to explain the genetic loading that may play a greater role than in the late-onset forms.

## Materials and Methods

### Patient Participation

Patients with EOS or EOB who were hospitalized in the Child and Adolescent Psychiatry Department, University Medical Center Maribor, Slovenia, and in the Adolescent Psychiatry Department, University Psychiatric Clinic Ljubljana, Slovenia, from January 2010 till December 2017, were recruited to the study. The DSM-IV-TR (for patients diagnosed before 2013) and DSM-5 (for patients diagnosed after 2013) diagnostic criteria for schizophrenia or bipolar disorder were used. A further inclusion criterion was early-onset disorder, thus only patients with the onset of the disorder before the age of 18 years were included. Medical or neurological disorder or IQ under 70 were exclusion criteria. All parents were offered genetic testing to determine whether genetic variants were inherited or occurred *de novo*. The protocol, which is consistent with the Declaration of Helsinki and its successive revisions, was approved by the Commission of the Republic of Slovenia for Medical Ethics (KME No. 103/11/12). After a complete description and comprehensive explanation of the procedures, the purpose of the study, and reassurance of confidentiality, written informed consent was obtained from the patients or their legal guardians. Each patient and their guardian were told that they were free to withdraw from the trial without any negative effect on their treatment. All the participants or their parents accordingly provided written informed consent.

### Genetic Analysis

Molecular karyotyping (Array-CGH) of genomic DNA from the patients was performed using Agilent SurePrint G3 Unrestricted CGH 8x60K (55.077 unique probes along the whole Human genome included). Confirmation of the CNVs were validated with a 4x180K (170.334 unique probes along the whole Human genome included) arrays, Agilent Technologies, Inc., NCBI build GRCh37. Analyzes were performed with two software tools BlueFuse Multi v3.3 Blue Gnome and Agilent Cytogenomics Software v4.0. Analysis settings were: 3 or more probes and Minimum Avg. Absolute Log Ratio 0.25/-0.25 were considered as duplications/deletions. Aberrations below 41 kb were not reported. The results were filtered to remove common CNVs (population frequency >1%) present in the Database of Genomic Variants, and in-house databases. CNVs were classified as benign/likely benign (bCNV), pathogenic/likely pathogenic (pCNV), or variants of unknown significance (VUS), according to the standards and guidelines of the American College of Medical Genetics (23). For Patient 7, karyotyping was used to confirm the array result.

## Results

Out of 98 invited patients who met the study criteria, 53 (54.0%) refused to take part because of the need for blood sample examination, a negative opinion of genetic analysis, or the logistics. Thirty-eight patients with EOS and seven with EOB were included in the study. The average age at the onset of EOS was 15.9 and 15.8 years in patients with EOB. The group with EOS comprised 25 boys and 13 girls, and the group with EOB included three boys and four girls (Supplementary Table 1). The detailed clinical picture with the age of onset, family history, and pharmacotherapy is given in Table 1.

**Table 1:** Clinical information of patients 1–7 containing DSM-5 diagnosis, main clinical symptoms, and age of onset and pharmacotherapy with results of molecular karyotyping according to the ISCN nomenclature, size of the deletion/duplication, encompassed genes, and CNV classification.

**Patient**	**Sex**	**DSM-5 diagnosis**	**Age of onset in years**	**Main clinical findings**	**Family history of mental or neurological disorders**	**Pharmacotherapy**	**ISCN nomenclature**	**Size (kbp)**	**CNV**	**Gene/syndrome**	**Clinical significance**
1	Male	EOS	16.5	Positive, disorganized, and negative symptoms	None	No significant improvement on maximal therapeutic dosages of risperidone, olanzapine, and clozapine	arr[GRCh37] 3q29(196534625_196599109)X1	64.5	del	*PAK2, SENP5*	VUS/likely pCNV
2	Male	EOS	13.5	Disorganized and negative symptoms	maternal grandfather and aunt: schizophrenia	Only a partial response to therapy with olanzapine but refused any therapy changes	arr[GRCh37] 15q25.2(82769959_84812664)X3	2042	dup	*TM6SF1, ADMTS3, SH3GL3, BNC1, BDTB1, FAM103A1, HOMER2, WHAMM, AP3B2, CPEB1, RPS17L*	pCNV
3	Female	EOB	17.0	With psychotic symptoms	father and her maternal grandfather: bipolar disorder	Stable on current therapy	arr[GRCh37] 15q13.2(30819487_31089960)X3	270.5	dup	*ARHGAP11B*	pCNV
4	Female	EOS	14.5	Mainly positive and cognitive symptoms	None	Included typical and atypical neuroleptics, mood stabilizers and benzodiazepines without full therapeutic effectiveness	arr[GRCh37] 9p22.2p22.1(17841908_18515947)X4~5	674	dup	*ADAMTSL1*	VUS
5	Male	EOS	15.5	Mainly positive symptoms	maternal uncle: suicide	Good therapeutic effect on atypical antipsychotic	arr[GRCh37] 16p11.2(29673984_30197316)X3 mat	523.3	dup	16p11.2 duplication syndrome	pCNV
6	Male	EOB	13.5	With psychotic symptoms	None	Good therapeutic effect on mood stabilizer	arr[GRCh37] 22q11.21(18706023_19010479)X1 pat	304.4	del	*DGCR6, PRODH, DGCR5*	VUS
7	Male	EOB	17.5	With psychotic and aggressive symptoms	None	Good therapeutic effect on mood stabilizer	arr[GRCh37] (X)x1,(Y)x2	/	/	/	pCNV

*ISCN, International System for Human Cytogenetic Nomenclature; kbp, kilobase pair; CNV, copy number variation; EOS, early-onset schizophrenia; EOB, early-onset bipolar disorder; mat, maternal; pat, paternal; del, deletion; dup, duplication; VUS, variant of unknown significance; pCNV, pathogenic CNV*.

Genetic testing of the parents was performed in two patients (Patients 5 and 6) to determine the origin of the CNV. Although asked several times, the parents of the other patients declined testing because they had a negative attitude toward blood or saliva sampling for genetic analysis, were unreachable or dead (both parents in Patient 3 and father in Patient 4).

### Genetic Analysis

The results of molecular karyotyping showed CNVs in four patients with EOS (Patients 1, 2, 4, and 5) and two with EOB (Patients 3 and 6), with an overall diagnostic yield of 13%. In one patient with EOB (Patient 7), a chromosomal aneuploidy was confirmed by classical karyotyping. The results for the patients are summarized in Table 1.

## Discussion

This is the first study on a Slovenian population with EOS and EOB. It confirms the important role of genomic structural variants in determining the genetic cause of these two severe mental disorders. We identified CNV abnormalities already known to be associated with psychiatric disorders in six out of 45 patients (13.3%) and one chromosomal aneuploidy. The incidence of CNV abnormalities is significantly higher than reported in the literature, where CNVs were identified in 2.4% of patients with adult-onset schizophrenia and 0.5% of patients with adult-onset bipolar disorder (24). There are several possible explanations for this discrepancy. Firstly, many studies show a higher rate of disease-related CNVs in early-onset mental disorders compared to adult-onset disorders (2, 5–7). Secondly, the bias in the patient recruited to our study as only hospitalized patients with EOS and EOB, who usually present with a more severe clinical picture (and hypothetically, a higher genetic burden), were invited, more than half of whom refused to participate. Thirdly, our study also encompassed syndromic patients where pathogenic CNVs are associated with psychiatric phenotypes with a broad range of penetrance, varying from 2 to 33% (25).

### The Role of CNV Affected Genes in the Etiology of Schizophrenia

#### The Role of the PAK2 Gene in EOS

**Figure 1:**
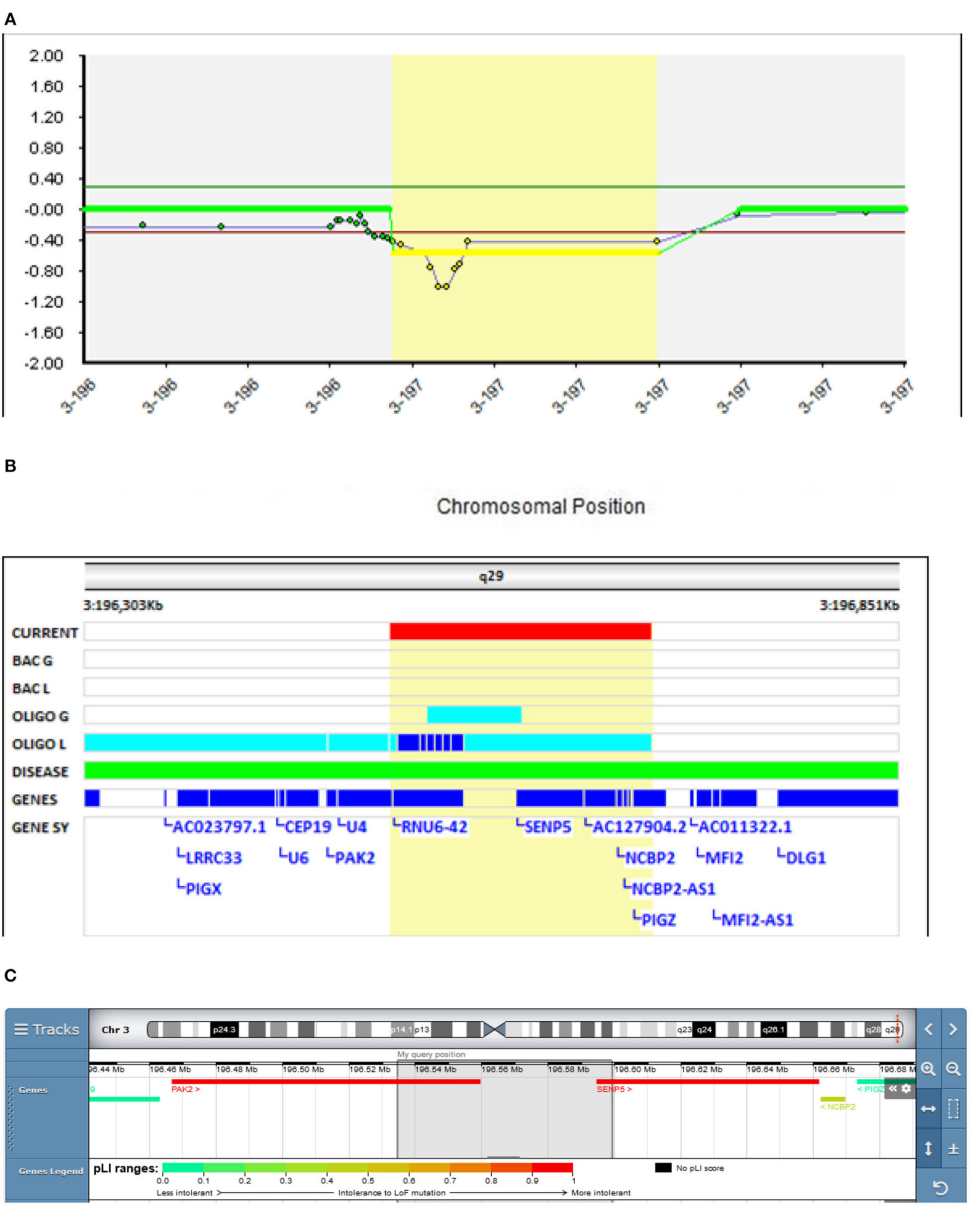
Molecular karyotyping of Patient 1 demonstrating deletion in 3q29 region, [GRCh37] 3q29(196534625_196599109)X1 **(A)**, which resulted in the partial deletion of the PAK2 and SENP5 genes **(B)**, according to UCSC Genome Browser **(C)**.

In our study, in Patient 1 (Decipher ID- 417759), who had severe schizophrenic symptoms from the age of 16.5 years and a chronic disease course, a deletion of 3q29 was found, which resulted in the partial deletion of the *PAK2* and *SENP5* genes (Figure 1). In the clinical presentation profound changes in his behavior, cognition and functioning were present. He was time disorientated, with disorganized thinking evidenced by disorganized speech including poverty of speech, illogicality and thought blocking. He had paranoid delusions, was distimic, and with auditory and visual hallucinations. Also negative (hypobulia, affective flattening) and cognitive symptoms were present. After several changes in antipsychotic therapy only partial remission was reached on clozapine. There was no history of psychiatric disorders in his family.

A meta-analysis done by Mulle et al. found that the 3q29 deletion may be the single largest risk factor for schizophrenia, surpassing even the 22q11.2 deletion (26). The 22 protein-coding genes in the 3q29 deletion interval deserve scrutiny as molecular targets that, when haploinsufficient, may underlie at least one form of schizophrenia. Several candidate genes have been implicated in the region, including *DLG1, PAK2*, and *FBXO45* (27). On the contrary, a study in mice showed that the *SENP5* gene is not a phenotypic driver gene in the 3q29 deletion syndrome (28), meaning that the *PAK2* gene plays an essential role in the observed deletion.

The serine/threonine kinase *PAK2* is a critical regulator of cytoskeleton dynamics. Synaptic cytoskeleton dysfunction represents a common pathogenesis in neurodevelopmental disorders, such as ASD. Wang et al. described *PAK2* haploinsufficiency resulting in markedly decreased synapse densities, defective long-term potentiation, and autism-related behaviors in mice (29). Evidence from post-mortem and large-scale genetic studies suggests that synaptic plasticity is disrupted in schizophrenia and may be a key pathogenic factor in the disease (30).

The microdeletion (64,485 bp) of 3q29 detected in Patient 1 with EOS is, to our knowledge, one of the smallest *PAK2* gene deletions reported in the literature, since ~20 genes are usually reported in the commonly deleted region (27). Therefore, our findings further support the schizophrenia-associated role of this gene. The Schizophrenia Working Group of the Psychiatric Genomics Consortium (PGC) (31) classified this CNV as a variant of unknown significance (VUS). We believe that its reclassification should be considered.

#### The Role of the ADAMTS3 Gene in EOS

**Figure 2:**
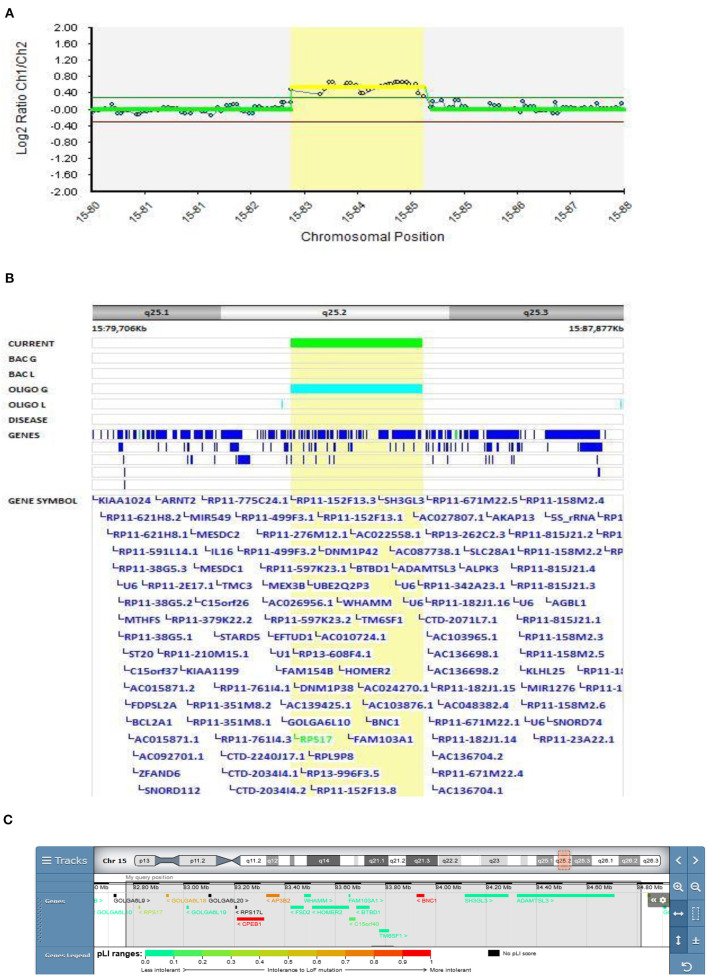
Molecular karyotyping of Patient 2 demonstrating duplication in 15q25.2 region, [GRCh37] 15q25.2(82769959_84812664)X3 **(A)**, encompasses 11 OMIM genes (Table 1), including *ADAMTS3* gene **(B)**, according to UCSC Genome Browser **(C)**.

In Patient 2 (Decipher ID – 417763), who had slowly progressive disorganized and negative schizophrenic symptomatology from the age of 13.5 years, a more than 2 Mbp 15q25.2 duplication was found (Figure 2), which was classified as pathogenic according to the CTD Gene-Disease Associations dataset (32). At the age of 13.5 Patient 2 presented with depression, social and school withdrawal. The response to the therapy with olanzapine was only partial but he and his family refused any kind of therapy change resulting in profound social isolation with cognitive and disorganized symptoms. History of schizophrenia was present in the family (mother's father and aunt).

CNVs mapping to 15q25.2 are implicated as a high-risk allele for pediatric neurological disease with variable outcomes as well as neuropsychiatric diseases such as schizophrenia and autism (33). Although clear pathogenicity for haploinsufficiency is described in ClinGen Clinical Genome Research (www.clinicalgenome.org), there is no strong evidence for dosage pathogenicity for triplosensitivity. In the DECIPHER v9.28 database (decipher.sanger.ac.uk), duplications coinciding or partially coinciding with the duplication in Patient 2 are defined as having mostly uncertain pathogenicity. The main feature described in these patients is intellectual disabilities, usually associated with other clinical features. A psychiatric condition has been reported in only two of the above-mentioned patients (288459 and 304631): psychosis with intellectual disability and behavioral abnormalities with cognitive impairment, respectively, but both patients have additional CNVs that could be involved in the development of their phenotype.

Patients 2's duplication encompasses 11 OMIM genes (Table 1), of which *RPS17, HOMER2*, and *ADAMTS3* have already been described as schizophrenia-associated genes in the literature (34–36). The *ADAMTS3* gene is the only gene overlapping the duplicated regions in our and DECIPHER patients 288459 and 304631, making this gene the most plausible candidate for psychiatric phenotypes. A study of ADAMTS3-knock-out mice also showed that inhibition of ADAMTS3 may be effective in the treatment of neuropsychiatric and neurodegenerative disorders (37), suggesting that duplication of this gene should be further studied to exclude its opposite effect. Our finding also further emphasizes the corroborating evidence of the putative involvement of the 15q25.2 duplication in EOS.

#### The Role of the ADAMTSL1 Gene in EOS

**Figure 3:**
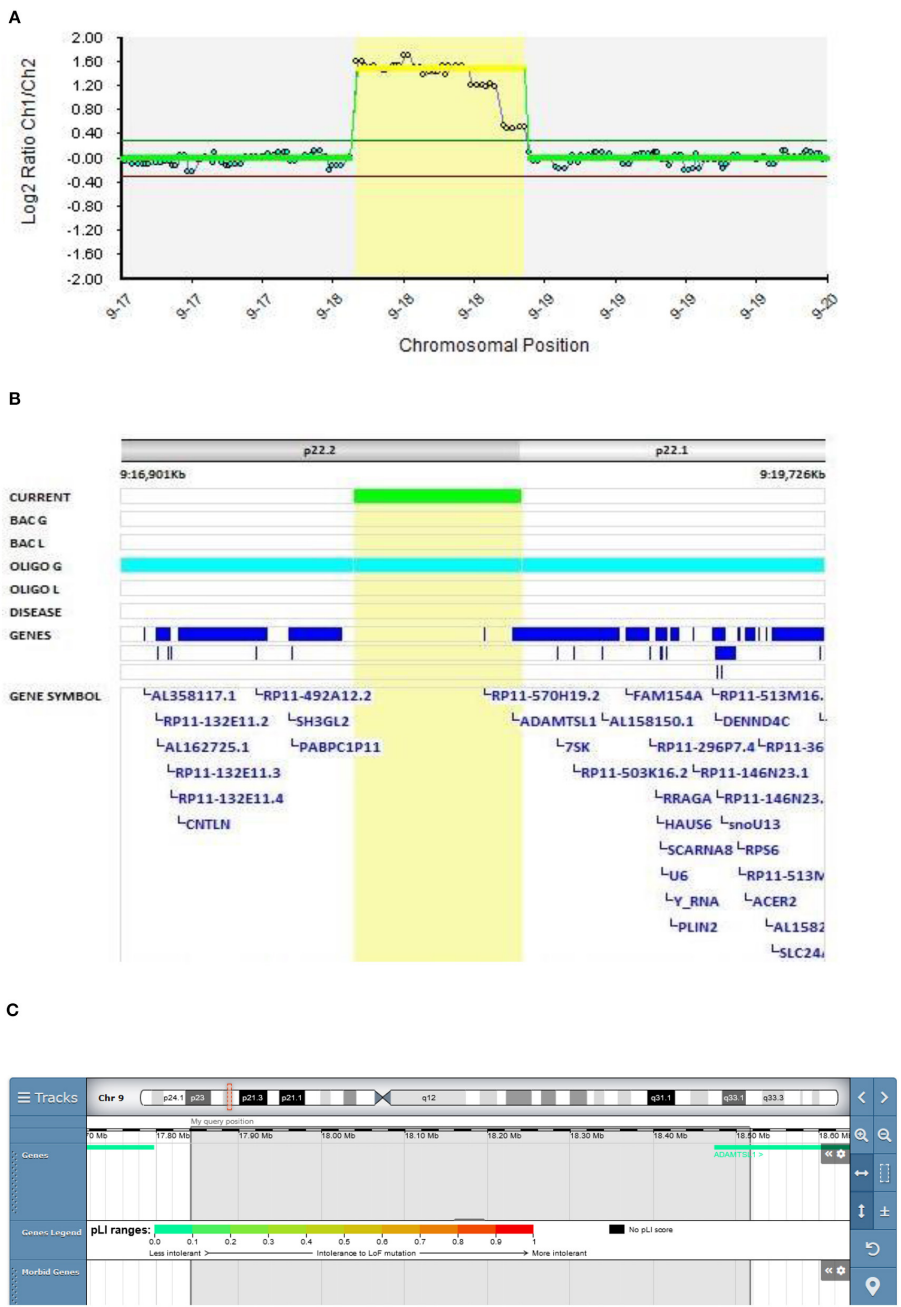
Molecular karyotyping of Patient 4 demonstrating multiplication in 9p22 region, [GRCh37]9p22.2p22.1(17841908_18515947)X4~5 **(A)**, encompassing *ADAMTSL1* gene **(B)**, according to UCSC Genome Browser **(C)**.

In Patient 4 (Decipher ID – 417766), with EOS diagnosed at the age of 14.5 years, the 9p22.2 region of 674 kb was multiplicated 4 to 5 times. The multiplicated region is part of the 9p duplication syndrome and partially captures the *ADAMTSL1* gene (Figure 3). Patient 4 was born with prominent feature of the short neck. Her first hospitalization at the adolescent psychiatry department was at the age of 14.5 because of depersonalization, paranoid delusions, and cognitive symptoms with poor concentration. Her speech was monotonous and her affect was flat. Afterwards she was hospitalized several times because of worsening of schizophrenic symptoms. Her pharmacotherapy included typical and atypical antipsychotics, mood stabilizers, and benzodiazepines but no full therapeutic effect was ever reached. Her father died from heart attack. Her mother was very peculiar in behavior and thinking, ambivalent to the pharmacotherapy of her daughter, sometimes actively rejecting any medication. Along with ambivalence to pharmacotherapy, mother had also paranoid ideas about laboratory, genetic, and neuropsychological testing which resembled delusions. Following her paranoid believes mother refused to be involved in any kind of medical testing including genetic.

9p duplication carriers present with microcephaly/brachycephaly, down-slanting palpebral fissures, bulbous nasal tip, short philtrum, and a short neck (38–40). Only the short neck was present in our patient. However, great clinical heterogeneity is observed depending on the 9p genomic regions involved in the rearrangements (41).

ADAMTS enzymes are metalloendopeptidases that have a broad range of functions in development and diseases due to their extracellular matrix remodeling activity in pathophysiological remodeling, and in inflammation and vascular biology (42).

Only one patient with a 9p duplication of the *ADAMTSL1* gene partly covering the same region as Patient 4 is described in the DECIPHER v9.28 database. The patient was reported to have a clinical picture of abnormal fear/anxiety-related behavior, autistic behavior, and global developmental delay. The pathogenicity is described as unknown (Patient 277935). In the study of Tangsuwansri et al., the association of the *ADAMTSL1* gene with neuromuscular diseases was proposed (43). Hendee et al. found an association of the *ADAMTSL1* gene with a complex phenotype that does not encompass psychotic symptoms in a three-generational family (44). To our knowledge, we present the first description of *ADAMTSL1* gene duplication in a patient with EOS.

#### The Role of 16p11.2 Microduplication in EOS

**Figure 4:**
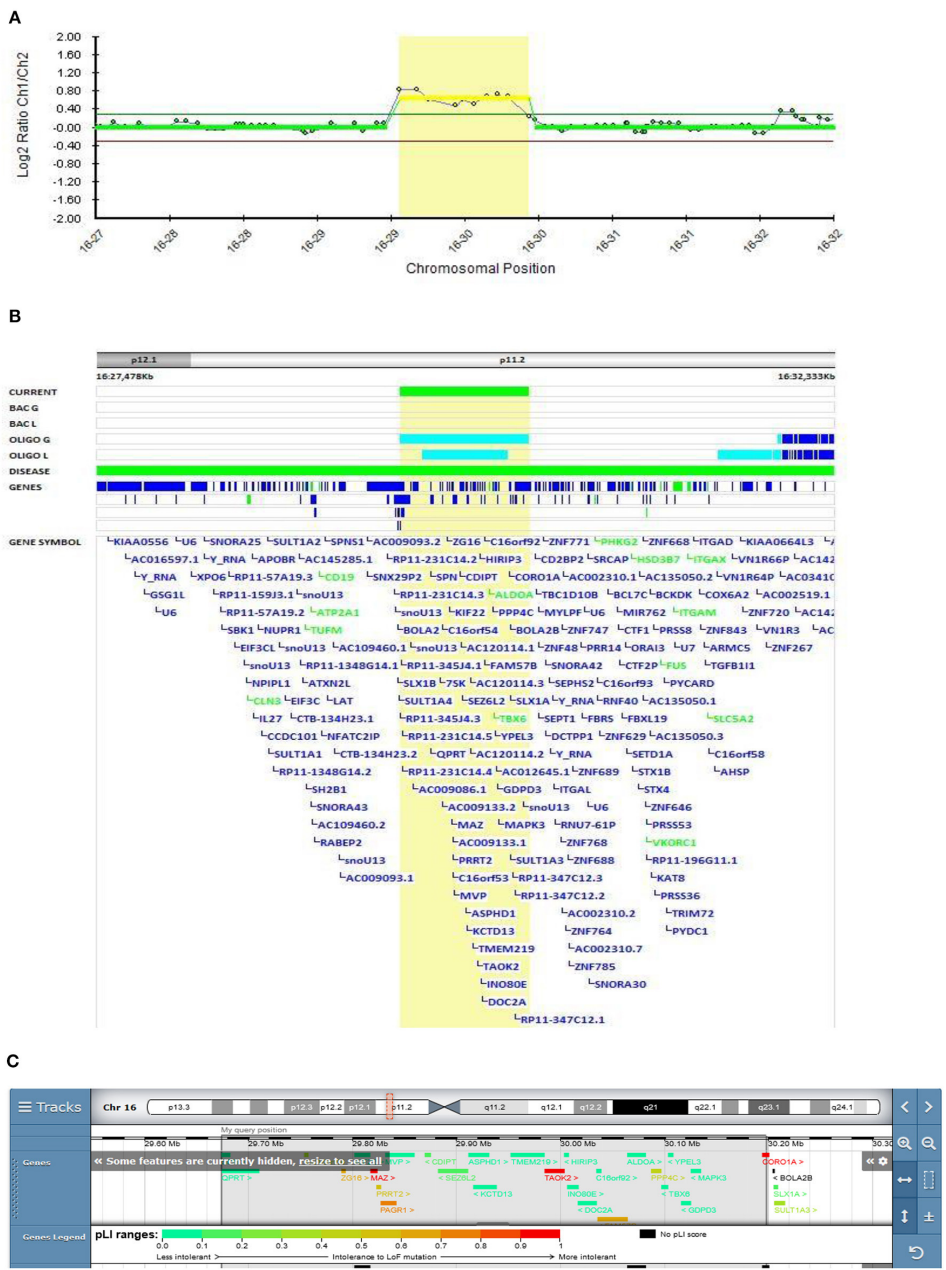
Molecular karyotyping of Patient 5 demonstrating duplication in 16p11.2 region, [GRCh37] 16p11.2(29673984_30197316)X3 **(A)**, which included the recurrent breakpoint (BP) regions BP4 and BP5 **(B)**, according to UCSC Genome Browser **(C)**.

In Patient 5 (Decipher ID – 417768), in whom EOS started at the age of 15.5 years, a maternally inherited duplication that included the recurrent breakpoint (BP) regions BP4 and BP5, located in the proximal region of 16p11.2, was found (Figure 4). Patient 5 presented with positive psychotic symptoms at the age of 15.5. He described auditory hallucinations, paranoid and depressive delusions and was confused, paramimic, parathymic and with thought blocking. He responded on the treatment with olanzepine with good therapeutic effect. There was a history of suicide in the family (mother's uncle). His mother could be described as specific in her behavior and thinking. She was silent, shy and social withdrawn but she did not fulfilled diagnostic criteria for any mental disorder.

According to the literature, the 16p11.2 BP4-BP5 duplication is the CNV most frequently associated with ASD, schizophrenia, and comorbidities such as decreased body mass index (BMI) (45). This variant was classified as pathogenic according to the Schizophrenia Working Group of the PGC publication. Two large cohorts with 16q11.2 deletion and duplication have been described by Steinman et al. as the most frequent genetic etiologies of ASD and other neurodevelopmental disorders (46). The mean effect of the duplication on cognition is similar to that of the reciprocal deletion, but the variance in the duplication is significantly higher, with severe and mild subgroups not observed with the deletion. These results suggest that additional genetic and familial factors contribute to this variability. The frequency of schizophrenia in the duplication cohort was lower than reported in the study by McCarthy et al., where a 14.5-fold increased risk of schizophrenia was observed (47). Their results suggest that the microduplication of 16p11.2 confers a substantial risk of schizophrenia and other psychiatric disorders, which is in accordance with our finding.

### The Role of CNV- Affected Genes in the Etiology of Bipolar Disorders

#### The Role of the ARHGAP11B Gene in EOB

**Figure 5:**
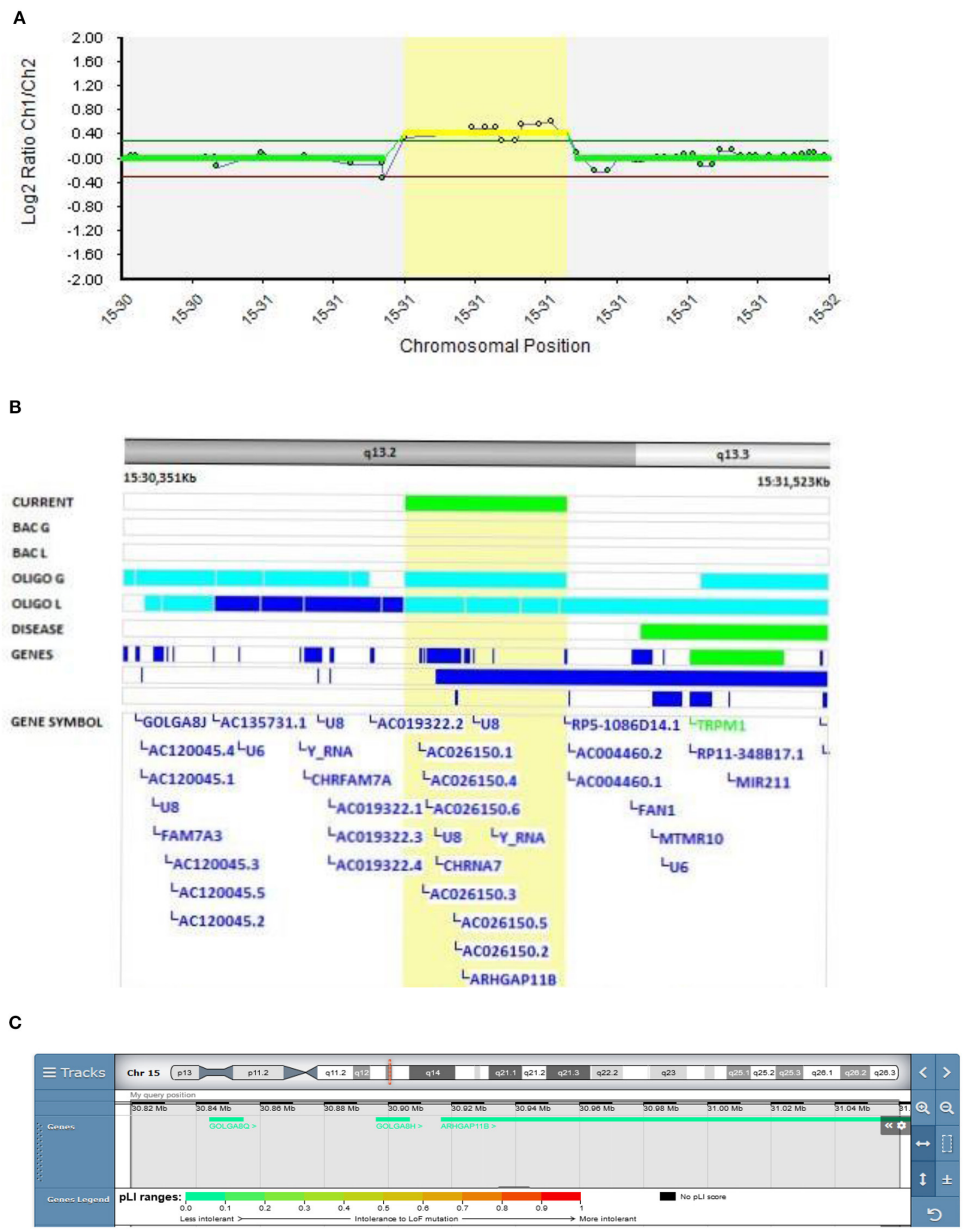
Molecular karyotyping of Patient 3 demonstrating duplication in 15q13.2 region, [GRCh37] 15q13.2(30819487_31089960)X3 **(A)**, encompassing *ARHGAP11B* gene **(B)**, according to UCSC Genome Browser **(C)**.

A 15q13.3 duplication encompassing the *ARHGAP11B* gene was found in Patient 3 (Decipher ID – 417765)with EOB (Figure 5). At the age of 17, Patient 3 was admitted to the adolescent psychiatry department due to profound changes in her behavior presented as social isolation, feelings of depersonalization, and severely depressed mood with paranoid delusions. After 2 months of treatment with antipsychotic and antidepressant therapy she had a first manic switch, followed by an ultra-rapid cycling clinical course of bipolar disorder. She was stabilized on the combination of lithium, clozapine, aripiprazole and sodium valproate. There was a history of bipolar disorder in her family (her father and mother's father).

A review of the literature found reports of 241 deletions and 79 duplications of the 15q13.3 region, with incomplete penetrance and variable expressivity mediating adult-onset schizophrenia (48).

Rho GTPase-activating protein 11B (ARHGAP11B) is one of the key factors that regulate neocortical expansion in the human brain (49). Although there is no clear correlation between the size of the neocortex and cognitive abilities, studies suggest that expansion of the neocortex in the human brain is the basis for our higher cognitive abilities compared to other species (50). Furthermore, a pilot study of CNVs on the data set from a total of 3,414 patients with schizophrenia, 713 patients with bipolar disorder, and 3,215 controls, showed that CNVs affecting 15q13.2 (*ARHGAP11B* and *FAN1* genes) appeared to be schizophrenia-specific (3).

In the vicinity (200 kb proximal and distal) of the observed duplication, the *CHRFAM7A* and *FAN1* genes are located. The *CHRFAM7A* gene is a chimeric product of the *CHRNA7* gene, which is genetically linked to multiple disorders with cognitive deficits, including schizophrenia and bipolar disorder. *CHRFAM7A* mRNA represents ~10–20% of the α7 sequence in the mRNA of the human brain (51). Long-distance regulation of both *CHRNA7* and *CHRFAM7A* has been reported. Duplication in the vicinity of the *CHRFAM7A* gene could interfere with the gene regulation. Chromosome conformation capture on chip (4C) analysis was utilized to show genomic interaction of the DNA sequence at chromosome 15q11.2 with both *CHRFAM7A* and *CHRNA7* at 15q13.3 (52).

On the other hand, a study of the *FAN1* gene suggests that this could be a driver gene in the 15q13.3 locus for psychiatric disorders since this gene encodes DNA repair enzyme, thus abnormalities in DNA repair could lead to susceptibility to SCZ or ASD (53).

15q13.2 microduplication, including the *ARHGAP11B* gene, was classified as probably pathogenic according to the Schizophrenia Working Group of the PGC publication. Therefore, we hypothesize that 15q13.3 duplication in the *ARHGAP11B* gene and possible deregulation of the *CHRFAM7A* and *FAN1* genes are associated with schizophrenic symptoms in EOB patients.

#### The Role of the PRODH Gene in EOB

In Patient 6 (Decipher ID – 417769), who was diagnosed with EOB at the age of 13.5 years, a 22q11.2 deletion was found to be inherited from a healthy father. It is known that the 22q11.2 deletion syndrome (DS) is linked to congenital anomalies and to a 20-fold increased risk of developing schizophrenia (54, 55). Patient 6 was first hospitalized at the adolescent psychiatry department because of positive psychotic symptoms at the age of 13.5 and his first psychiatric diagnosis was psychosis. His first manic symptoms presented as elevated mood, logorea, grandiose delusions, and diminished need for sleep a year after. Clear depressive episode revealed 4 months after first manic episode. Mood stabilizator lithium was introduced resulting in full remission. There was no history of psychiatric disorders in his family. His father was never diagnosed with psychiatric disorder and there was nothing special or peculiar neither in his behavior or thinking.

**Figure 6:**
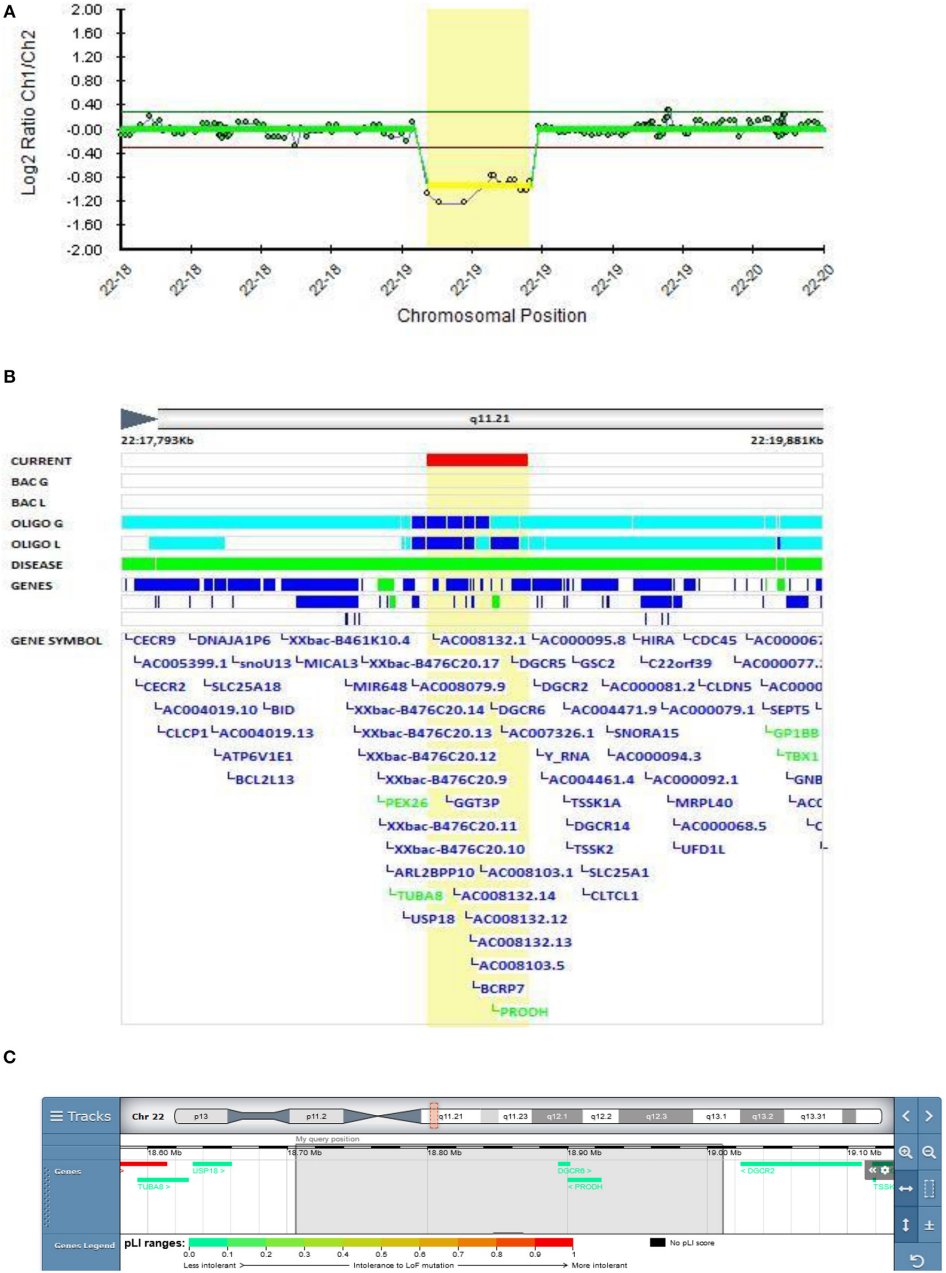
Molecular karyotyping of patient 6 demonstrating deletion in 22q11.21 region,[GRCh37] 22q11.21(18706023_19010479)X1 **(A)**, encompasses the *DGCR6, PRODH* genes, and the first four exons of the *DGCR5* gene **(B)**, according to UCSC Genome Browser **(C)**.

In Patient 6 in the present study, the deletion is located proximal to 22q11.2 DS and encompasses the *DGCR6* and *PRODH* genes, and the first four exons of the *DGCR5* gene (Figure 6). This variant was classified as VUS according to the Schizophrenia Working Group of the PGC. The father has the same deletion encompassing the same three genes (DGCR6, PRODH, and the first four exons of DGCR5 genes).

Meng et al. identified a potential role for *DGCR5* in regulating certain schizophrenia-related genes (56). Significantly lower *DGCR6* expression was found in children with 22q11 DS who had anxiety disorders (57). Mutations in the *PRODH* gene coding proline dehydrogenase are thought to be linked to behavioral alterations in schizophrenia, but the role of *PRODH* in their etiology remains unclear. In the Zwarts et al. study, knockdown and overexpression of the slgA gene, the Drosophila *PRODH* homolog, was observed in the mutant Drosophila brain, which led to altered aggressive behavior, thus confirming the role of proline metabolism in the etiology of increased aggression (58). A study on 1402 participants with 22q11.2 DS, aged 6–68 years, showed that attention deficit hyperactivity disorder (ADHD) is the most frequent disorder in children with 22q11.2 DS (37.1%). In adolescents with 22q11.2 DS, anxiety disorders were more prevalent, and psychotic disorders were present in 41% of adults over the age of 25 with 22q11.2 DS. Bipolar disorders increased with age, but the occurrence was low, even in adults (59). Toma et al. observed deletions encompassing the *PRODH* gene in several bipolar patients in extended families, which supports our finding and provides corroborating evidence of the putative involvement of the *PRODH* gene in bipolar disorder (5).

#### The Role of Chromosomal Aneuploidy 47 XYY in EOB

**Figure 7:**
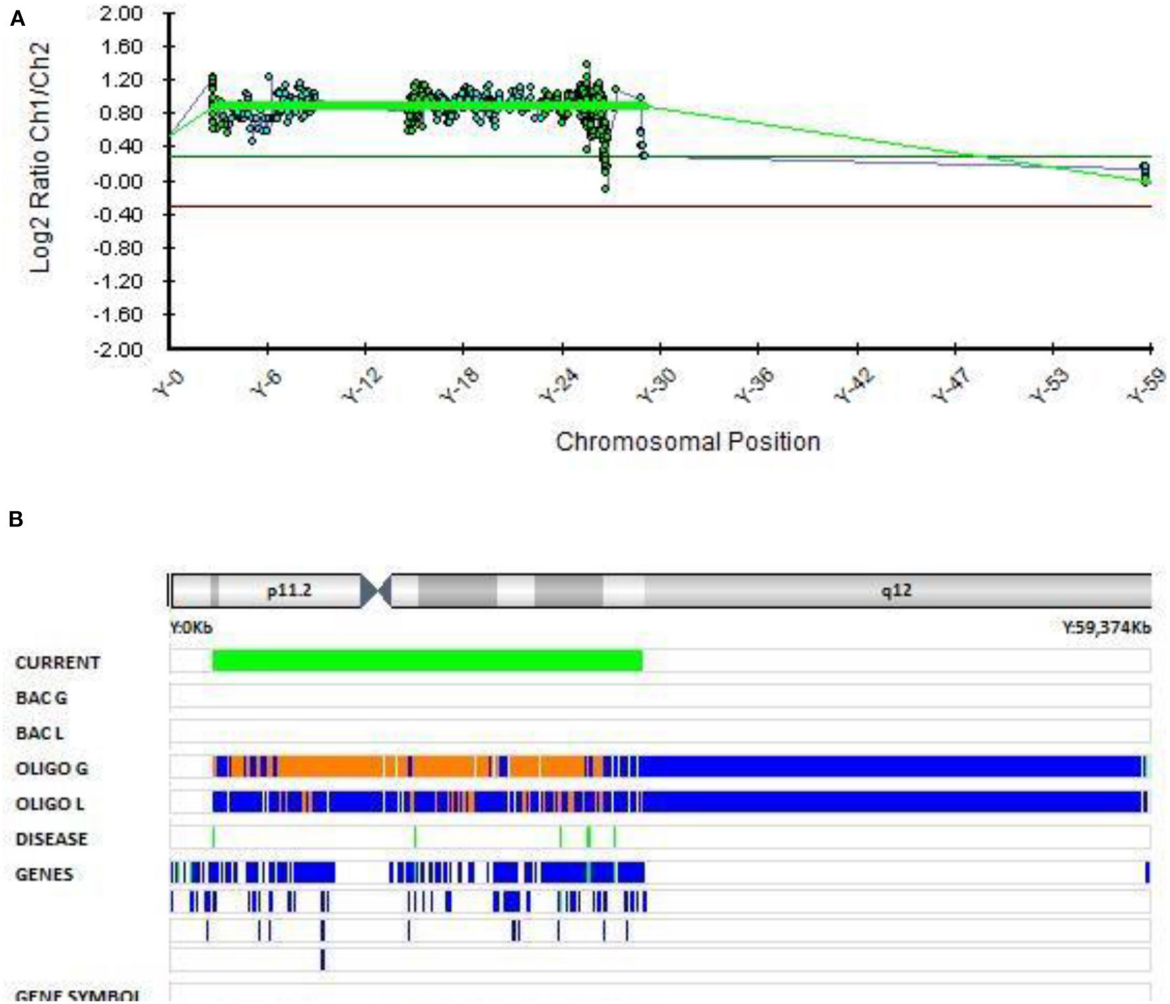
Molecular karyotyping of patient 7 demonstrating complete duplication of chromosome Y,[GRCh37] (X)x1,(Y)x2 **(A,B)**, which resulted in 47,XYY karyotype.

In Patient 7 with EOB, an additional copy of the Y chromosome was found with molecular karyotyping (Figure 7). This chromosome rearrangement was classified as pathogenic according to the Schizophrenia Working Group of the PGC publication. Patient 7 was first hospitalized at the adolescent psychiatry department at the age of 17.5 because of manic, psychotic and aggressive symptomatology. He presented with aggressive out bursts, inappropriate behavior, impulsivity, grandiose delusions about his superpower and ability of control the people and events and irritable affect. He was stabilized on the therapy with quetiapine and risperidone with no major relapses. There was no history of psychiatric disorders in his family.

The presence of an extra Y chromosome has been associated with neurodevelopmental disorders (ASD, schizophrenia, and ADHD) in many studies (60–65). ASD can occur in as many as 40% of individuals with an extra Y chromosome (66). However, sex chromosome aneuploidies in mood disorders have been poorly investigated (67). A Danish study from 2001 of sex chromosome aneuploidies in patients with schizophrenia or bipolar disorder reported no evidence of increased risk for bipolar disorder or schizophrenia with aneuploidies of the X or Y chromosomes. However, they found a significant increase in the occurrence of the combined phenotype, including both bipolar disorder and schizophrenia, in individuals with the 47, XYY karyotype (68). It is rare for schizophrenia and bipolar disorder to be described in one phenotype and we could not locate any further studies that found an increased risk of EOB in patients with 47, XYY karyotype. Thus, this is one of the few reports of the 47, XYY karyotype in a patient with EOB.

### Genetics and Pharmacotherapy

Treatment resistance to antipsychotics was observed in three out of seven patients (42.8%) with CNVs (Patients 1, 2, and 4) (Table 1). This concurs with the study of Kushima et al. who found that the rate of treatment resistance to antipsychotics was significantly higher in patients with CNVs than in those without (36.1 vs. 16.9%, odds ratio = 2.79, *P* = 0.0036) (69). Therefore, CNV findings may be useful in predicting the response to antipsychotics in patients with schizophrenia.

### Research Limitation and Future Directions

The limitations of the study were its small sample size and the lack of either a control group or an adult-onset group for comparison. The small sample size was expected given the rarity of true early- onset schizophrenia and bipolar disorder. There was also some inclusion bias since only hospitalized patients were invited to participate in the study, more than half of whom declined. In cases of rare, generally small CNVs, parental testing is recommended, since it is important for proper interpretation of the detected variant. Additional family member testing in inherited CNV cases is advisable when a specific phenotype segregates within the family (70). In our study, most of the patients' parents did not participate in genetic testing, although they were invited several times. Therefore, precise clinical classification of the observed CNVs was not possible, which is one of the main limitations of the study. We recommend further studies with a larger sample size, an appropriate control group and family genetic testing of all patients if possible.

The main strength of the present study is in the inclusion of patients with early-onset mental disorders, which are rarer and not as frequently studied as those with adult-onset. Early onset of the disease could mean a greater role for the genes involved in disease development. Our study supports the findings from the literature regarding the association of the *PAK2, ARHGAP11B*, and *PRODH* genes with schizophrenia and/or bipolar disorder. We also present the first report of a duplication of the *ADAMTSL1* gene and the smallest deletion of the *PAK2* gene in patients with EOS, and one of the few reports of the 47, XYY karyotype in a patient with EOB. Although 108 Schizophrenia-Associated Genetic Loci have been identified and specific genes pinpointed (71, 72), much still has to be done in terms of identifying the precise role of the specific gene or microdeletions and microduplications encompassing several genes in the etiology of schizophrenia and bipolar disorders. Thus, further studies like ours are still needed.

## Conclusion

We conducted the first analysis of genomic CNVs in a study of 45 patients with EOS and EOB in Slovenia. Our findings support the association of the *PAK2, ARHGAP11B*, and *PRODH* genes with these two disorders. We also present the first report of a duplication of the *ADAMTSL1* gene in a patient with EOS and one of the few reports of the 47, XYY karyotype in a patient with EOB. Our observed rate of CNVs is significantly higher than reported in the literature, which could be the consequence of a higher genetic contribution in early-onset disease, the small number of cases observed, and additional recruitment bias. We observed a high treatment resistance to antipsychotics in patients with CNVs.

## Data Availability Statement

The datasets presented in this study can be found in online repositories. The names of the repository/repositories and accession number(s) can be found below: Decipher database, IDs: 417759, 417763, 417765, 417766, 417768, and 417769.

## Ethics Statement

The studies involving human participants were reviewed and approved by Commission of the Republic of Slovenia for Medical Ethics (KME No. 103/11/12). Written informed consent to participate in this study was provided by the participants' legal guardian/next of kin.

## Author Contributions

DK wrote the manuscript, performed genetic analysis and data interpretation, reviewed the article and revisions, and gave final approval of the article. NK wrote the manuscript, performed genetic analysis and data interpretation, reviewed the article and revisions, and gave final approval of the article. MD enrolled the patients and collected and assembled the data. NS enrolled the patients and collected and assembled the data. AZ performed the genetic analyses. HG designed the study, wrote the manuscript, interpreted the data, reviewed the article and revisions, and gave final approval of the article. All authors contributed to the article and approved the submitted version.

## Conflict of Interest

The authors declare that the research was conducted in the absence of any commercial or financial relationships that could be construed as a potential conflict of interest.
